# COVID-19 Incidence and Death Rates in the Southern Region of the United States: A Racial and Ethnic Association

**DOI:** 10.3390/ijerph192113990

**Published:** 2022-10-27

**Authors:** Luma Akil, Yalanda M. Barner, Anamika Bisht, Ebele Okoye, Hafiz Anwar Ahmad

**Affiliations:** 1 Department of Behavioral and Environmental Health, School of Public Health, College of Health Science, Jackson State University, Jackson, MS 39217, USA; 2 Department of Health Policy and Management, School of Public Health, College of Health Science, Jackson State University, Jackson, MS 39217, USA; 3 Department of Epidemiology and Biostatistics, School of Public Health, College of Health Science, Jackson State University, Jackson, MS 39217, USA; 4 Department of Biology, College of Science, Engineering, and Technology, Jackson State University, Jackson, MS 39217, USA

**Keywords:** COVID-19, southern USA, health disparities, minority population, socioeconomic

## Abstract

The SARS-CoV-2 virus responsible for the COVID-19 pandemic continues to spread worldwide, with over half a billion cases linked to over 6 million deaths globally. COVID-19 has impacted populations unequally based on income, age, race, sex, and geographical location. This study aimed to characterize COVID-19 incidence and death rate trends in six states of the southern region of the USA and to understand the demographic and racial differences in its incidence and death rates. Data for the study were collected from the COVID-19 Data tracker of the Centers for Disease Control and Prevention for the following southern states: Alabama (AL), Florida (FL), Georgia (GA), Louisiana (LA), Mississippi (MS), and Tennessee (TN). The results showed a significant geographical variation in the COVID-19 cases and related deaths. Significant variations in COVID-19 cases and death rates were observed among different races and ethnic groups. The highest number of COVID-19 cases were observed among the Hispanic and Black populations, and the highest death rates were found among non-Hispanic Blacks and Whites. The southern states included in this paper showed a high number of COVID-19 cases and high death rates during the study period. These increased rates may result from the low socioeconomic status and large minority populations.

## 1. Introduction

The World Health Organization (WHO) declared the outbreak of the infectious coronavirus disease 2019 (COVID-19), originating in Wuhan (China), a pandemic on 11 March 2020 [1]. The incidence and death rates of COVID-19 have been overwhelming globally, nationally, and statewide in the USA. The virus that causes COVID-19 is thought to spread mainly from person to person through respiratory droplets produced from coughs, sneezes, or talks by the infected person [2,3]; however, research has shown that infection is spread mainly among those individuals who have not been diagnosed with the disease [4].

As of February 2022, more than 390 million cases and 5.7 million deaths linked to COVID-19 have been reported globally [1]. In the United States (US) alone, over 7 million cases and 800 thousand deaths were reported [1]. In 2020, the US emerged with the highest COVID-19 case counts and mortality rates globally [4]. The first documented case of COVID-19 in the US was detected in the West region of the country, but the virus rapidly spread to other US areas. Several measures, policies, and regulations were imposed in the states including the closure of businesses, mask mandates, physical distancing, etc., to help in mitigating COVID-19 and reducing the mortality rates [5].

High incidences of COVID-19 and death rates were reported in the southern region of the US. [6] The southern region of the US accounts for more than one-third of the US population, including high minority populations and approximately 19% of the Black/African American residents in the south, compared with 8% who reside in the other regions of the US [7,8]. Studies have suggested that the rates of COVID-19 are elevated among Blacks and Hispanics [6], in addition to increased rates of COVID-19-related hospitalization, ICU admission, and death [9,10,11]. Minority populations may be at a higher risk of infectious disease outbreaks due to socioeconomic factors and comorbidities such as obesity, diabetes, cardiovascular diseases, and others. Such disparity results in higher infectious disease incidences and deaths [10,12,13]. The Centers for Disease Control and Prevention reported that individuals with complications of diabetes, obesity, fear-related disorders, and anxiety are more likely to be at a greater risk of death related to COVID-19 [3].

Socially disadvantaged states and countries with large racial and ethnic minority populations and communities of color were disproportionately affected by COVID-19 and shown to have elevated COVID-19 infection and death rates [14,15]. Socioeconomic inequality plays a pivotal role in how communities are affected. People of lower socioeconomic status, including education, income, or employment, suffer from a lower state of health than those of higher socioeconomic status. The influencing socioeconomic factors include living in deprived areas; working in high-exposure or frontline occupations; living in large, multigenerational households; a high burden of underlying conditions; discrimination; and poor access to health or community services [9]. In addition, socioeconomic hardships can impede the access to healthcare and adversely affect individuals’ health, translating into elevated mortality rates and the burden of chronic diseases [16].

Incidence and death rates of COVID-19 have, at times, overwhelmed the local hospitals’ capacity to provide their usual care protocols. Studies suggested that Black populations have a higher risk for hospitalization due to SARS-CoV-2 than White populations [17]. Further, a recent study found that 34.6% of Black children were exposed to COVID-19 compared to 11.3% of White children [18].

In this study, we examined COVID-19 incidence and death rates over a two-year period in six southern states with similar socioeconomic and health disparities status, to determine the trend and changes in the selected southern states and to highlight the changes in demographics and race over that time period.

Thus, in this project, we aimed (1) to determine the trend of COVID-19 incidence and death rates in selected states of the southern region of the US, including Alabama, Florida, Georgia, Louisiana, Mississippi, and Tennessee from March 2020 through February 2022 and (2) to understand the demographic, socioeconomic, and racial differences in COVID-19incidence and death rates.

## 2. Materials and Methods

Data for the study were collected from the CDC COVID-19 Data tracker [19] available at: https://covid.cdc.gov/covid-data-tracker/#datatracker-home (accessed on 18 March 2022). The data collected for the study included the COVID-19 Weekly Cases and Deaths per 100,000, Population by Age, Race/Ethnicity, and Sex from 23 January 2020 to 28 February 2022, for the following selected southern states: Alabama (AL), Florida (FL), Georgia (GA), Louisiana (LA.), Mississippi (MS) and Tennessee (TN) are available at: https://covid.cdc.gov/covid-data-tracker/#demographicsovertime (accessed on 18 March 2022) [20]. However, for MS, the data were only available for 2020 and not for 2021 and 2022, so MS data on COVID-19 incidence and death rates in relation to race and ethnicity were collected from the Mississippi State Department of Health (MSDH) [21]. This study focused on six southern states with similar socioeconomic status. These states were also selected as a representative of the southern region of the US. In addition, 7-Day Case Rates per 100,000 and 7-Day Death Rates per 100,000 were collected for each state and are available at: https://covid.cdc.gov/covid-data-tracker/#trends_dailycases (accessed on 18 March 2022) [20]. Socioeconomic data including poverty rate, unemployment rate, percent of uninsured, percent of Medicaid-insured, percent of high school education and percent of college education were collected form the Bureau of Labor Statistics, the US Department of Agriculture, and the Kaiser Family Foundation [22,23,24]. In addition, the racial percent of the selected states populations was collected form the US Census Bureau’s world population review [25].

Data were analyzed using SPSS v 28 [26]. Descriptive and frequency analysis and analysis of variance (ANOVA) were carried out to determine the significant differences between the states, age groups, sex, race, and ethnicity. Simple linear regression analyses were carried out to determine the trend over time and the unit change in COVID-19 cases and death and to understand their seasonality and fluctuation over time. The model used time as an independent variable and COVID-19 cases and death rates as dependent/response variables. Correlational and regression analyses were also conducted to determine the association between COVID-19 incidence and death rates with socioeconomic factors such as poverty, unemployment, education level, uninsured rates, and insurance through Medicaid

Further, GIS maps were created using the ArcGIS software package (ESRI: Redlands, CA, USA; V 10.8.2) [27]. Maps were created for the COVID-19 cases and death rates based on the racial differences in the selected states. GIS maps helped visualize the geographical content and understand relationships and patterns in the study areas.

### 2.1. GIS Mapping

ArcMap is a main component of the desktop ArcGIS suite of the geographic information system (GIS) application that supports viewing, editing, printing, and geospatial data analysis.

### 2.2. Study Area for the GIS Map

Located in the southern US, AL (32.3182° N, 86.9023° W), FL (27.6648° N, 81.5158° W), GA (32.1656° N, 82.9001° W), LA (34.0522° N, 118.2437° W), MS (32.9906° N, 89.5261° W), and TN (35.5175° N, 86.5804° W) were selected for this study.

They cover a total area of 320,591 square miles. GIS allows for integrating and analyzing geographic data, such as coordinates and size perimeters, and tabular data (i.e., attributes of geographic data points). Data were imported into mapping software in layers, where each layer represents a different visual component of the map. States data were pooled, and maps’ layers for COVID-19 cases and deaths were created for the White, Black, and Hispanic populations to visually analyze areas with higher disease and death rates in relation to race.

## 3. Results

**Figure 1:**
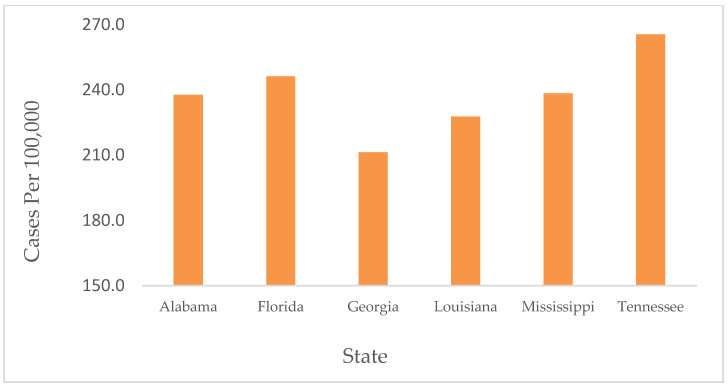
Average cases of COVID-19 by state from January 2020 through February 2022. Highest numbers of COVID-19 cases were observed in TN.

A total of 13,511,590 cases and 180,075 deaths were reported in the selected states during the study period between March 2020 and February 2022. A significant difference in number of cases and death rates was noted between the states (*p* < 0.05). The results showed the highest incidence of COVID-19 in TN, with a weekly average of 265/100,000. The lowest rate was in GA, with a weekly average of 211/100,000 cases (Figure 1).

**Figure 2:**
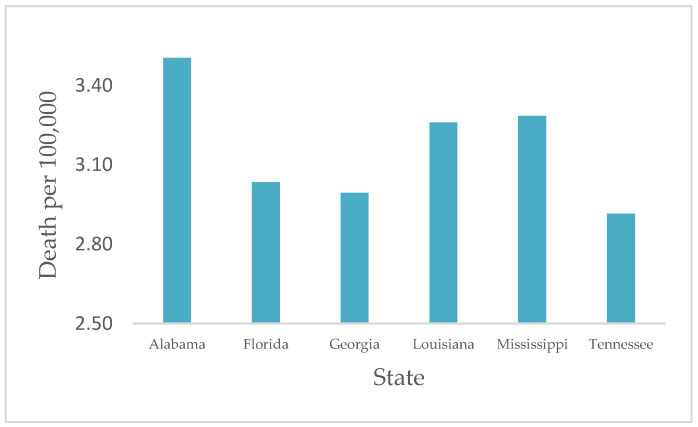
Average death rate by state from January 2020 through February 2022. The highest overall death rate was observed in AL.

Furthermore, a significant difference in COVID-19 death rates was observed between the selected states (*p* < 0.05). The death rates were the highest in AL, with a weekly average of 3.5/100,000 deaths, followed by MS and LA with 3.20/100,000 and 3.17/100,000 deaths during the study period (Figure 2).

**Figure 3:**
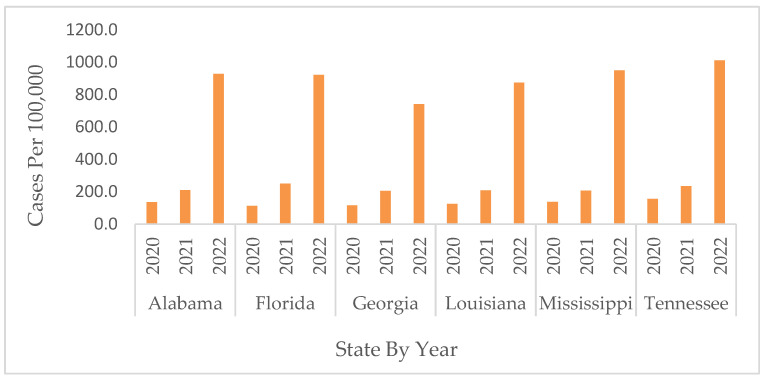
Average numbers of cases of COVID-19 per state from March 2020 through February 2022.

**Figure 4:**
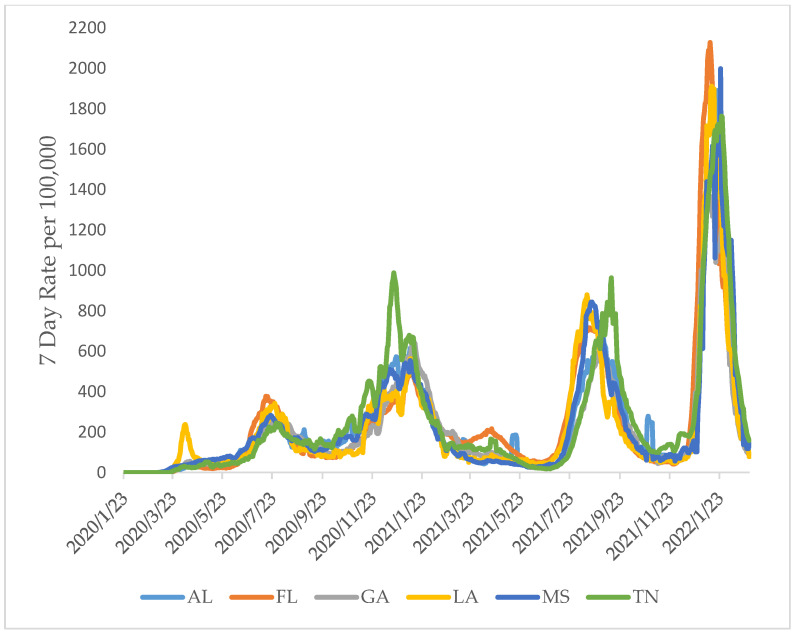
Time series analysis showing changes in the number of COVID-19 cases and related waves in the selected states from January 2020 through February 2022.

A significant increase in cases and death rates was observed from 2020 to 2022, with the highest rates in 2022 (Figure 3) (only January and February were included in this analysis for 2022). During the early stage of the pandemic in 2020, LA showed the highest number of cases of COVID-19, with a peak of 233/100,000 cases. However, by late 2020 and early 2021, TN showed the highest weekly average of cases, with a peak of 987.85/100,000 cases. Yet, by late 2021 and early 2022, the largest wave of COVID-19 cases impacted the study’s states, with the highest peaks in FL and MS, corresponding to 2127.58/100,000 and 1998.05/100,000 weekly average cases, respectively (Figure 4).

**Figure 5:**
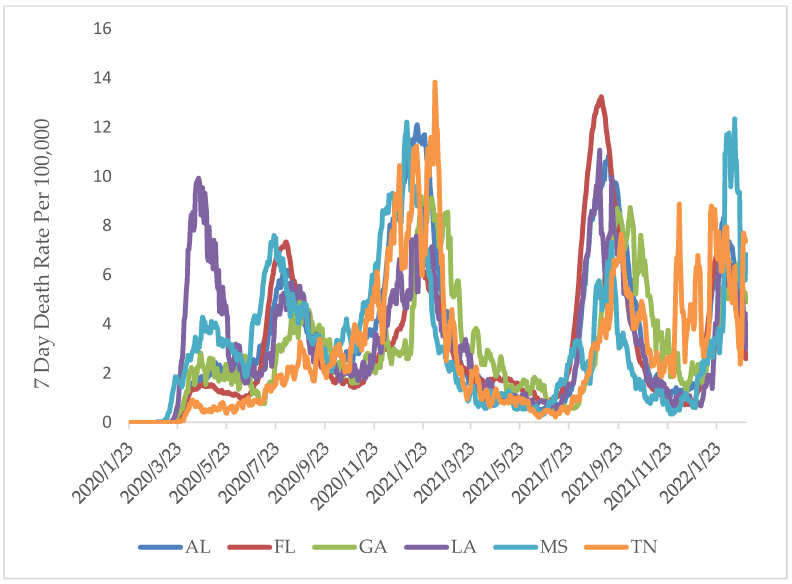
Time series analysis showing COVID-19 death rate change over time in the selected states from January 2020 through February 2022.

A significant variation in COVID-19 death rate was also observed between the states and over time between 2020 and 2022 (*p* < 0.05). LA showed the highest death rate during the early stages of the pandemic, with the highest rate of 9.81/100,000. However, a shift in death rates was observed by early 2021, with TN and MS showing the highest death rates (13.82/100,000 and 12.2/100,000 weekly averages, respectively). During Fall of 2021, FL witnessed the highest death rates from COVID-19, with a peak rate of 13.06/100,000, while MS showed the highest death rates during the early 2022 with 12.33/100,000 average weekly death rate (Figure 5). A regression analysis was carried out, with COVID-19 cases and deaths as dependent variables and time as an independent variable. The correlation coefficient (r = 0.45) indicated a moderate to substantial increase in cases over time and a moderate (r = 0.235) increase in deaths over time. In addition, the corresponding coefficients of determination (R^2^_cases_ = 0.202; R^2^_deaths_ = 0.055) showed the unit change over time for the cases and death rates, respectively.

The results from this study showed no significant difference in the cases or death rates between males and females in all states during the study period, as shown in Table 1.

**Figure 6:**
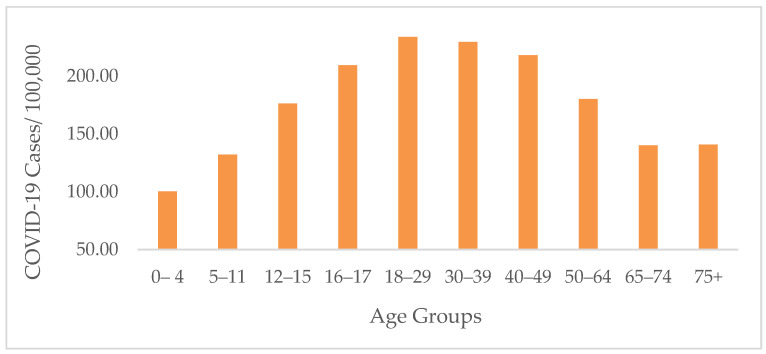
Average COVID-19 cases by age group in all states from 2020 to 2022. The highest rates were found among the 18–29- and the 30–39-year age groups.

**Figure 7:**
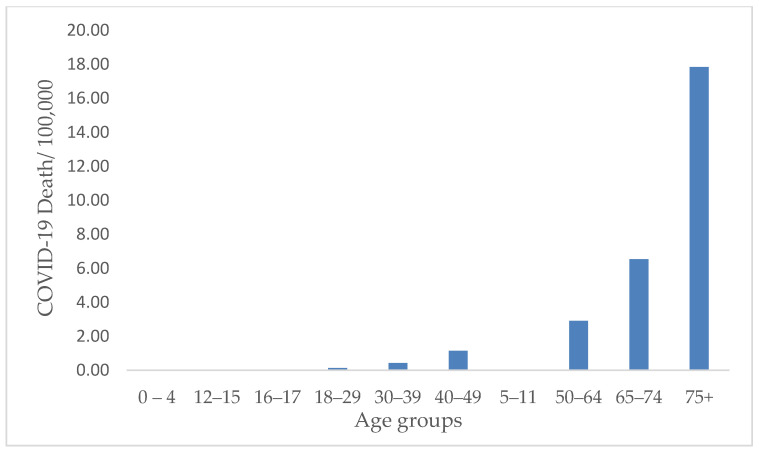
Average COVID-19 death rate by age group in all states from 2020 to 2022. The highest death rate was observed in the elderly population above the age of 75 years in the selected states.

COVID-19 cases and death rates varied significantly in different age groups (*p* < 0.001). The highest rates of COVID-19 were among the 19–28- and the 29–38-year age groups, with a seven-day average of 233.94 ± 308.35 cases/100,000 and 229.69 ± 310.50 cases/100,000, respectively (Figure 6). The highest rates of COVID-19 were in TN among the 19–28-year age group, with an average of 359.13/100,000 cases (Table 2). However, the death rate from COVID-19 was the highest among the elderly group of 75-year-olds and older, with an average of 17.83/per 100,000 deaths during the study period (Figure 7). TN showed the highest death rates among the 75-year-old and older populations, with a seven-day average of 23.75/100,000 death (Table 2).

**Figure 8:**
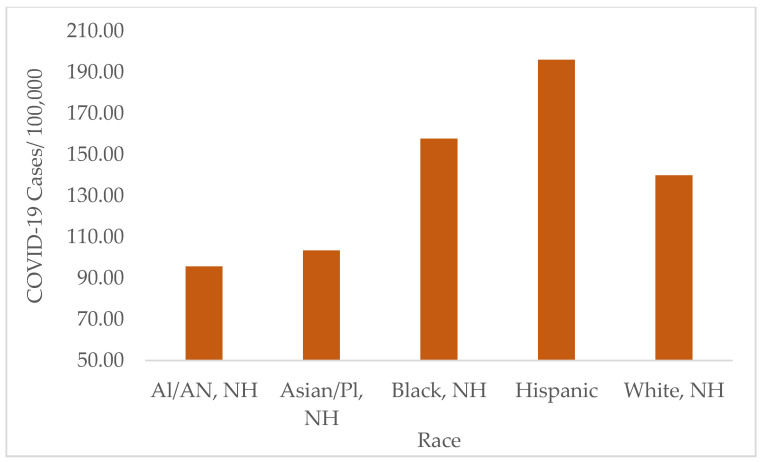
Average number of COVID-19 cases by race in all states from 2020 to 2022. The highest number of COVID-19 cases was observed in the Hispanic population, followed by the Black/African American population (*p* < 0.001). Note: AI: American Indian, AN: Alaska Native, NH: Non-Hispanic, Pl: Pacific Islander.

A significant variation in COVID-19 cases and death rates was observed between different races and ethnicities in the selected states (*p* < 0.01). The highest number of COVID-19 cases were observed among Hispanics, which was significantly different from that in all non-Hispanic ethnicities, with a weekly average of 196.15 ± 304.03/100,000 cases (*p* < 0.01). Black/African American populations also showed high rates of COVID-19 cases, with a weekly average of 157.77 ± 233.00/100,000 cases. The numbers of Black/African American cases were not significantly different from that of White cases, whose weekly average was 139.95 ± 188.56/100,000 cases (*p* > 0.05). Furthermore, no significant difference in the COVID-19 rates was observed between American Indian/Alaska Native and Asians/Pacific Islander groups (*p* > 0.05). These groups had the lowest rates of COVID-19 cases, which were significantly different from those in other races (*p* < 0.05) (Figure 8).

**Figure 9:**
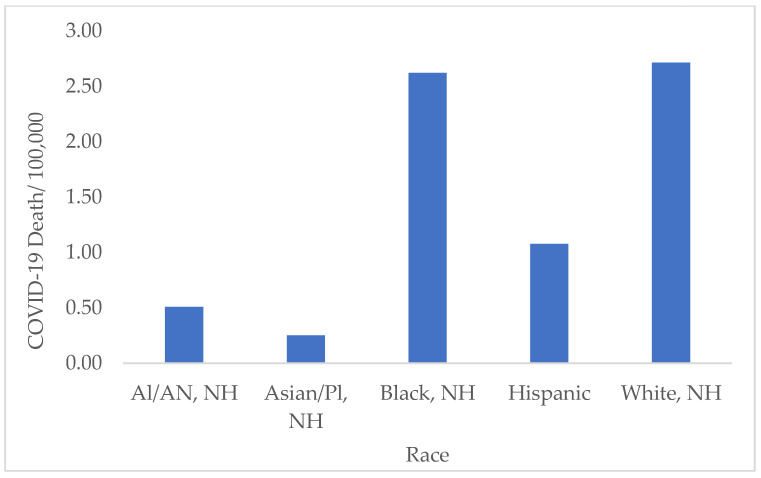
Average rate of COVID-19 deaths by race in all states from 2020 to 2022. The highest death rates were observed among the White and Black/African American Populations. Note: AI: American Indian, AN: Alaska Native, NH: Non-Hispanic, Pl: Pacific Islander.

The death rates were significantly different between the different racial groups in the selected states. However, there was no significant difference in the death rates between the White and Black populations and between American Indians/Alaska Natives and Asians/Pacific Islanders (*p* > 0.05). The highest death rates were observed in the White and Black/African American populations, with a weekly average of 2.71 ± 4.89 2.67/100,000 and 2.62± 2.76/100,000 deaths, respectively (Figure 9).

**Figure 10:**
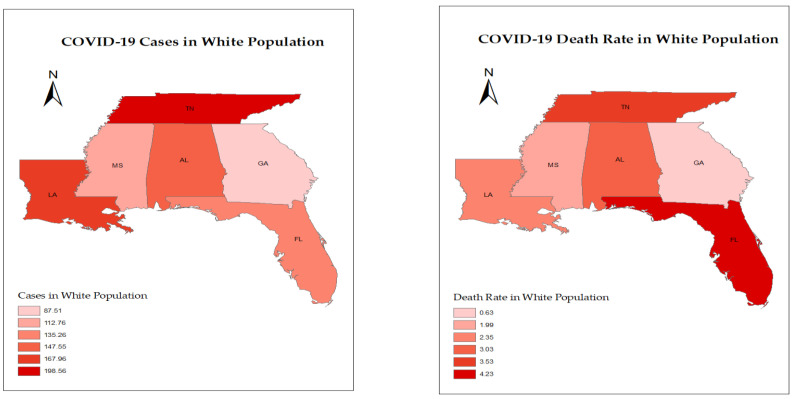

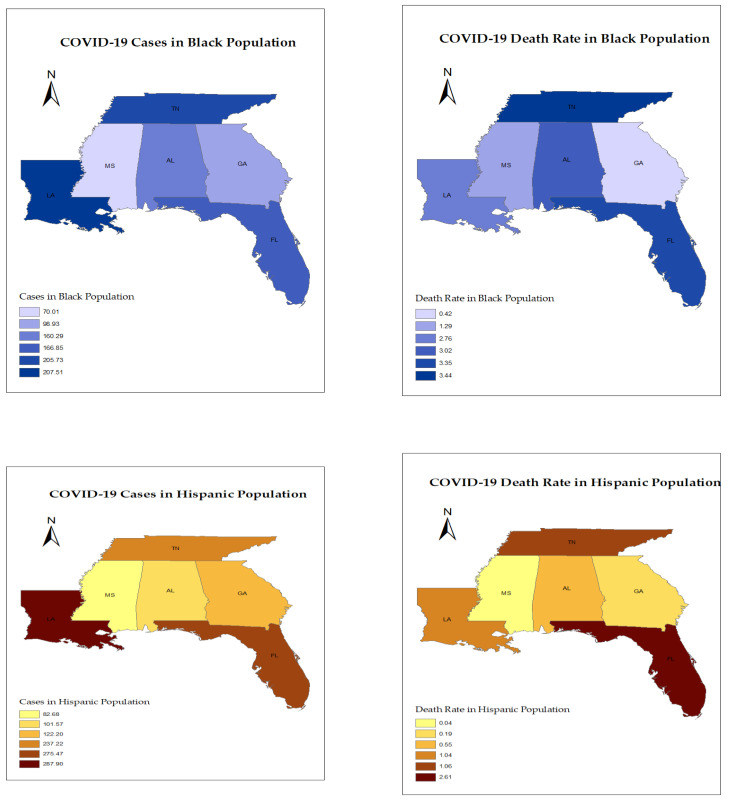

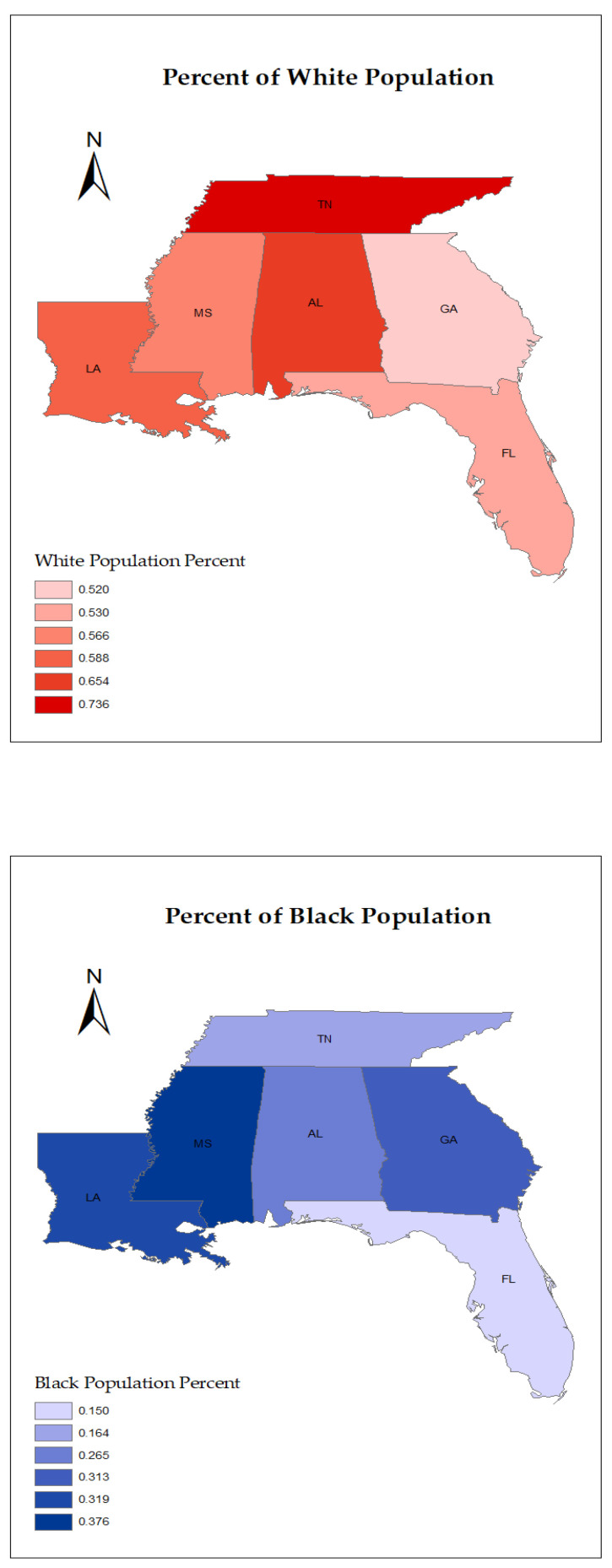

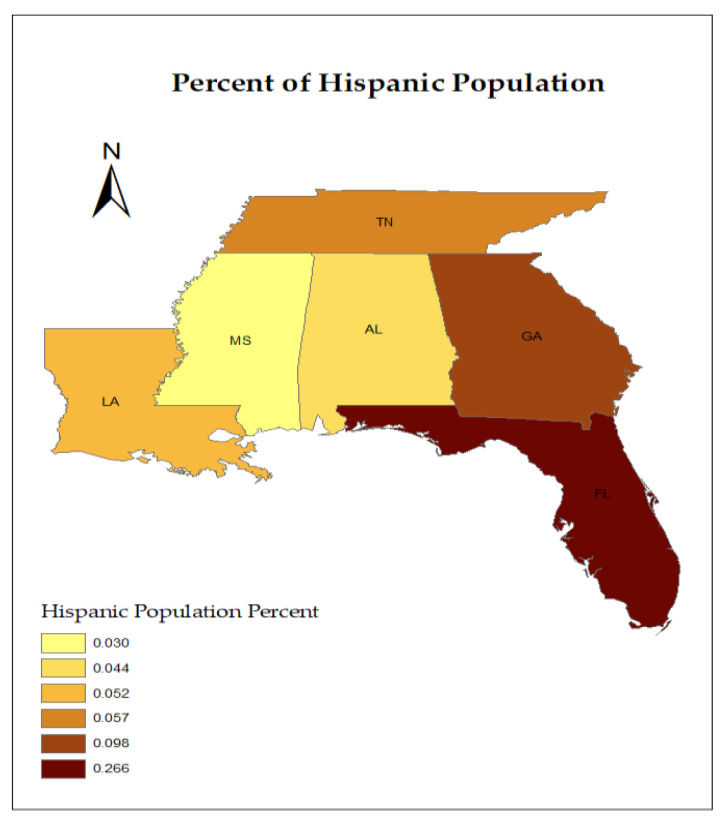
GIS Maps of COVID-19 cases and death rates in the selected states from March 2020 through February 2022.

The highest number of COVID-19 cases was observed in the Hispanic population in FL and LA, with a weekly average of 275.47/100,000 and 287.90/100,000 respectively, while Blacks/African Americans reported the highest number of cases in AL, with a weekly average of 160.29/100,000 cases, and in MS, the White population contributed to the high number of cases of COVID-19 with a weekly average of 112.76/100,000. The highest death rate was observed among the White and Black/African American populations in AL, FL, GA, MS, and TN, while, in LA, the highest death rate was observed among the Black/African American populations, with 2.76/100,000 weekly average. The lowest rates of COVID-19 cases were observed among the American Indians/Alaska Natives, followed by the Asian and Non-Hispanic Pacific Islander groups, with a weekly average of 95.74/100,000, 103.50/100,000 cases, respectively, while Asians and Non-Hispanic Pacific Islanders reported the lowest death rate, with a weekly average of 0.25/100,000 and 0.50/100,000 for the American Indian and Alaska Native groups. LA showed the highest number of cases, while FL had the highest death rates for these groups (Table 3). The GIS maps are shown in Figure 10 to visualize the racial differences related to the number of COVID-19 cases and death rate in the selected states. Races and ethnicities with the highest rates are only shown on the maps.

Socioeconomic variables varied significantly between the study’s states. All states had a poverty rate higher than the US average of 12.3%, with MS having the highest poverty rate of 19.07%. All states, except for LA, had an uninsured percentage higher than the US average, with LA, MS, and TN having a higher percentage of Medicaid-insured individuals compared with the US average. The unemployment rates were the highest in MS. All states’ high school education rates were higher than the US average, except for MS, and all states reported a higher college education than the US average. In addition, most of the states had a higher percent of Black/African American population, except AL and GA, compared with the US average, with the highest in TN; the highest rate of Hispanic population was in GA (Table 4).

Regression and correlational analyses were carried out, with COVID-19 cases and deaths as dependent variables and socioeconomic variables as an independent variable. These regression models showed a week negative correlation between COVID-19 cases and poverty (y = −1.2264x + 256.76; R^2^ = 0.0384), a week positive correlation between COVID-19 cases and unemployment (y = 9.1717x + 209.35; R^2^ = 0.0514), and a positive moderate correlation with high school education (y = 1.9621x + 163.61; R^2^ = 0.1389). However, the death rates resulting from COVID-19 were shown to be positively associated with poverty (y = 0.0428x + 2.5029; R^2^ = 0.3102) and negatively associated with high school education (y = −0.0332x + 4.4202; R^2^ = 0.2642).

## 4. Discussion

The southern region of the United States has witnessed significantly higher COVID-19 cases and death rates since the beginning of the pandemic, compared to the rest of the country. Our study observed the highest rates in TN, FL, MS, and AL. All states, except GA, had a higher case average compared with the US average of 215/100,000 cases during the study period. AL, MS, and LA had the highest death rates, while all studied states reported a higher death rate compared with the US weekly average of 2.54/100,000. These high rates of COVID-19 cases and deaths in the studied states are due to political policies, high percentages of minority populations such as Black/African Americans and Hispanics, a low socioeconomic status, and a low access to health care.

All of the studied southern states are controlled by the Republican Party, except for GA that became controlled by the Democratic Party during the 2020 elections. During the early months of the pandemic, all southern states’ governments issued a “Safer at Home” order. This order included social distancing guidelines, rules on gathering capacities, closing schools and universities or switching to online learning, mask mandates, and vaccination starting in early 2021 [28,29,30,31,32,33,34]. Studies have shown that the majority of people complied with the guidelines; however, the individual responses to the pandemic were related to their political affiliation and beliefs [35]. Many people “did not perceive the threat of disease as severe and thus considered preventative measures excessive” [30]. In addition, several reasons affected people’s compliance with the preventive measures: “not believing it was necessary, distrust in science and/or authority, and belief in free choice.” In addition, many believed that “wearing a mask was harmful to breathing, a healthy diet will prevent COVID-19, and that God will protect them” [36]. These perceptions were evident in the Southern region of the US. In addition, most of these states allowed an earlier activity resumption compared with other states that were controlled by the Democratic Party; many Republicans were also less likely to engage in behaviors such as wearing masks or face coverings than Democrats, suggesting radical disparities in health practices, split along political fault lines [35]. Researchers also suggested that policymakers should assist in reducing social interaction and making practical choices [37].

In this study, the highest numbers of COVID-19 cases were found among the young adults and mid-aged groups of 18 and 49 years of age. These results are similar to those of other studies that analyzed age-specific COVID-19 morbidity and mortality data. Studies showed that working adults between 20 and 49 years of age have more contact with other adults and are more susceptible to COVID-19 than younger individuals [38]. In addition, prioritizing vaccination to older populations over 60 years of age and those with comorbidities to reduce fatalities in these populations may have impacted the spread of the virus among individuals under 50 years of age, especially in disadvantaged populations [39].

In addition, our study showed that the death rates were higher among patients over 65 years of age. COVID-19 patients older than 50 years had a 15-fold higher mortality risk than patients younger than 50 [40]. The older populations are more prone to infections due to the progressive decline of natural immunity. They are more vulnerable to adverse drug reactions because of reduced organ function or comorbidities [40]. As for the presence of older adults in the southern region of the US, FL has the highest population of adults over 65 years of age (21.1%), followed by AL (17.5%) [34]; the high number of older people has also contributed to the high mortality rates in the south.

Our study did not find a significant gender difference in COVID-19 incidence and death rates; however, females had higher rates of COVID-19 in all states. Males reported higher rates of death resulting from COVID-19 in all states, except for TN and MS. Previous studies showed that male patients with COVID-19 have a significantly increased mortality risk than female patients. Male patients aged ≥50 years or with comorbidities have a significantly increased mortality risk [40].

The highest numbers of cases of COVID-19 were found among the Hispanic and Black/African American populations, while the White and Black communities had the highest rates of death. The communities of racial minorities such as Blacks and Hispanics have been disproportionately affected by COVID-19 from the early stages of the pandemic [41]. Even though there was no significant difference in COVID-19 rate between Black/African American and White populations in this study, several studies demonstrated that Black and Hispanic individuals were more likely to test positive for COVID-19 than White individuals [10,11,12,13,17]. The studied southern states have a high population of Black, Hispanic, and other minority populations. About 26.5%, 15%, 31.3%, 31.9%, 37.6%, and 16.4% of the Black/African American population live in Al, FL, GA, LA, MS, and TN, respectively, compared with the average of 12% in the rest of the US [42].

In the studied southern states, the COVID-19 cases and death rates were the lowest among the American Indians, Alaska Natives, Asians, and Pacific Islanders. The percent of these populations is less than 1% in the selected states, so these rates were lower compared with those for other groups. However, in some studies, American Indian, Alaska Native, and Pacific Islander populations were shown to experience an excess mortality due to COVID-19, while Asian populations had similar rates of infection, hospitalization, and death as White populations [17]. Moreover, Asian patients had the highest risk-adjusted cardiorespiratory disease severity when hospitalized [43].

In addition, studies have found that minority ethnic groups have higher risks of COVID-19-related hospitalization, ICU admission, and death [9,13,43,44]. One study found that Hispanic and Black patients comprised more than half of the patients hospitalized with COVID-19; these patients were more often uninsured, had a lower socioeconomic status, and experienced longer delays from symptom onset to COVID-19 diagnosis [43]. Another study in Louisiana found that non-Hispanic Black individuals represented 77% of the hospitalized patients and 71% of the deaths, despite only constituting the 31% of the total population [44]. Other factors such as living in deprived areas, working in high-exposure or front-line occupations, and living in large, multigenerational households may increase the risk of COVID-19-related hospitalization, ICU admission, and death [9].

Minority populations suffer from high poverty, obesity, hypertension, cardiovascular disease, and diabetes and have low education and income levels, which are associated with a lower socioeconomic status [45]. In this study, a weak negative correlation was found between the number of COVID-19 cases and poverty, a weak positive correlation between the number of COVID-19 cases and unemployment, and a positive moderate correlation between the number of COVID-19 cases and high school education. In addition, the death rate resulting from COVID-19 was shown to be positively associated with poverty and a negatively associated with high school education.

Socioeconomic differences can impede shelter-in-place orders for minority populations such as Blacks or Hispanics who live in high-density housing, work as frontline employees, and live in food deserts, factors that do not allow for social distancing or self-isolation during infections to reduce the transmission of a virus [41,46]. Studies also showed that cities with better socioeconomic resources, which allowed for better infrastructure and response, were better at containing the virus [47]. Further, studies showed that poorer Black patients were at higher risk of death, mechanical ventilation, and ICU admission [46]. They also predicted that male sex, obesity, and low neighborhood median income were more significant predictors of poor outcomes for Black patients than for White patients. Minority children have a higher likelihood of contracting the SARS-CoV-2 infection. Non-Hispanic Blacks have an odds ratio for the risk of infection of 2.3, and Hispanics of 6.3 over non-Hispanic Whites [18].

Socioeconomic factors such as income, education, food security, and healthcare access, are strongly linked to the spread of COVID-19 and related death. Studies indicated that Black patients living in significantly poorer neighborhoods than White patients had lower educational attainment, a higher unemployment rate, and a higher poverty rate, were more often participants of the Supplemental Nutrition Assistance Program (SNAP), and were more likely to have Medicaid insurance. Hispanic populations also had difficulties avoiding public transportation, a low ability to telework, and a higher difficulty obtaining child are. It was also indicated that the racial disparities associated with the COVID-19 pandemic are systemic inequities rooted in structural racism [18,46]. In addition, the COVID-19 pandemic’s effects on rural populations have been severe, with significant negative impacts on unemployment, overall life satisfaction, mental health, and economic outlook [48]. Further, comorbidities such as diabetes, obesity, cardiovascular disease, asthma, and liver and kidney disease result in a high incidence of cases and death among minority populations [13,47].

Poverty is an indicator of the socioeconomic status. Even though our findings were not very significant, which may be due to the high poverty and low socioeconomic status in all of the studied states, other studies that examined the association of COVID-19 with poverty in several states with a variation of the socioeconomic status showed that a low socioeconomic standing increased the exposure to COVID-19. People with high poverty and a low socioeconomic status usually receive healthcare services at an advanced stage of illness and, thus, experience a worse prognosis [12].

In addition, studies have found that during the pandemic, individuals with a high school diploma or less are more likely to work as essential workers for jobs such as food services and less likely to telework or work in low-contact positions, which leads to increased contact and exposure to infectious diseases [11]. Another study also found that being older, being male, having a low (or no) individual income, having a low education level, not being married, and being born in a low- or middle-income country independently predict a higher risk of death from COVID-19 [49].

In addition to poverty and a low education level, access to health care is a major contributor to infectious diseases and related death. Most of the states in the southern region of the US rank low in regard to health care affordability, adult dental visits, adult wellness visits, child dental visits, child wellness visits, and health insurance enrollment. AL, GA, and MS, for example, rank 49, 48, and 47, respectively, in regard to health care affordability, while MS, FL, and GA rank 48, 47, and 46, respectively, in health insurance enrollment [50]. Previous studies showed that existing underlying barriers to accessing healthcare provide some explanation for the increased susceptibility to serious negative health outcomes of ethnic minority and migrant women in the UK [51]. Access to health care was shown to be determined by a person’s ability to use health services with ease and having confidence in treatment [52]. Further, people of low socioeconomic status present to healthcare services at a more advanced stage of illness, which will result in poorer health outcomes, also in relation to COVID-19 [52].

## 5. Conclusions

In this study, we examined the incidence and death rates of the COVID-19 pandemic for around two years in six southern states with a similar socioeconomic status and rates of minority population. Our findings provide important insights about the geographical variation in the rates of COVID-19 in the US and the impact of the demographic and socioeconomic status. Within the southern part of the US, high COVID-19 incidence and mortality from COVID-19 were observed. A low socioeconomic status and higher rates of minority populations may be the cause of such elevated rates. Higher rates of COVID-19 morbidity and mortality were significantly associated with minority populations of Blacks and Hispanics.

## 6. Limitations of the Study

This study was conducted through a very volatile phase of the pandemic in the southern US states, with their built-in socioeconomic and other variations, including geographical and climatic ones. Such variations, besides the varying nature of the infection, different strains of the virus at play, and insufficient time to stabilize the data, led to unavoidable variability and uncertainty of the data and, to some extent, to limitations of the study. In addition, missing data and combining data from different sources may add to the limitations of the study. However, despite these deficiencies, the present research provides a timely insight into the COVID-19 status of minority populations in these southern states, highlighting already preexisting disparities.

## Figures and Tables

**Table 1:** Rates of COVID-19 cases and death rates by sex in the selected states.

State	Sex	Weekly Average Cases/100,000	STD	Average 7 Day Death Rate/100,000	STD
AL	Female	256.31	333.01	3.10	2.84
	Male	212.86	263.53	3.84	3.55
FL	Female	151.28	143.18	8.49	42.17
	Male	138.92	131.39	3.64	3.18
GA	Female	203.62	256.70	0.49	0.62
	Male	170.83	205.52	0.65	0.81
LA	Female	260.02	350.32	2.73	2.38
	Male	217.18	280.32	8.48	45.94
MS	Female	49.73	46.48	3.43	6.72
	Male	39.27	37.19	3.06	3.73
TN	Female	297.92	381.62	15.03	113.93
	Male	275.31	334.54	4.57	7.52

**Table 2:** Rates of COVID-19 cases and deaths by age group in the selected states from January 2020 through February 2022.

State	Age Group	0–4	5–11	12–15	16–17	18–29	30–39	40–49	50–64	65–74	75+
AL	Cases/100,000	62.22	74.05	107.95	138.87	147.46	137.98	136.19	117.57	99.85	113.45
	Death/100,00	0.05	0.00	0.00	0.04	0.15	0.56	1.50	3.86	8.64	21.69
FL	Cases/100,000	73.89	113.86	135.57	159.39	195.85	197.06	180.72	143.73	97.82	93.87
	Death/100,00	0.02	0.01	0.02	0.06	0.21	0.48	1.55	2.84	6.10	18.88
GA	Cases/100,000	89.87	130.85	175.11	204.33	235.33	235.47	219.38	184.80	137.23	139.09
	Death/100,000	0.01	0.00	0.01	0.01	0.04	0.12	0.28	1.16	1.71	4.01
LA	Cases/100,000	138.91	163.21	231.03	269.25	296.22	291.24	288.64	232.59	193.25	186.67
	Death/100,000	0.01	0.00	0.02	0.02	0.13	0.47	1.17	3.17	7.82	21.73
MS	Cases/100,000	14.44	13.33	21.70	37.19	57.95	54.11	56.52	48.47	42.51	61.24
	Death/100,000	0.02	0.00	0.00	0.00	0.08	0.30	0.67	2.12	5.29	15.21
TN	Cases/100,000	167.57	221.72	288.37	338.63	359.13	350.86	324.70	270.07	208.07	200.42
	Death/100,000	0.03	0.01	0.02	0.03	0.15	0.56	1.40	3.79	8.84	23.75

**Table 3:** Weekly mean and standard deviation for COVID-19 cases and death rates grouped by race and state.

State	Race	COVID-19 7-Day Average	Standard Deviation	COVID-19 7-Day Average Death	Standard Deviation
AL	Al/AN, NH	91.82	203.43	0.00	0.00
	Asian/Pl, NH	99.34	207.20	0.00	0.00
	Black, NH	160.29	250.28	3.02	2.51
	Hispanic	101.57	86.56	0.55	1.34
	White, NH	147.55	212.00	3.03	2.73
FL	Al/AN, NH	102.91	109.98	0.11	1.09
	Asian/Pl, NH	109.35	123.70	0.94	1.32
	Black, NH	166.85	156.22	3.35	3.29
	Hispanic	275.47	407.00	2.61	2.22
	White, NH	135.26	125.25	4.23	9.80
GA	Al/AN, NH	61.03	96.55	0.00	0.00
	Asian/Pl, NH	66.27	119.56	0.08	0.40
	Black, NH	98.93	151.01	0.42	0.59
	Hispanic	122.20	184.58	0.19	0.42
	White, NH	87.51	117.39	0.63	0.85
LA	Al/AN, NH	116.18	158.17	0.00	0.00
	Asian/Pl, NH	160.30	217.33	0.32	1.65
	Black, NH	207.51	317.59	2.76	2.85
	Hispanic	287.90	432.94	1.04	1.94
	White, NH	167.96	223.16	2.35	2.06
MS	Al/AN, NH	2.08	NA	0.01	NA
	Asian/Pl, NH	1.12	NA	0.03	NA
	Black, NH	70.01	NA	1.29	NA
	Hispanic	82.68	NA	0.04	NA
	White, NH	112.76	NA	1.99	NA
	Unknown	59.81	NA	0.53	NA
TN	Al/AN, NH	101.01	142.80	0.00	0.00
	Asian/Pl, NH	109.05	137.86	0.00	0.00
	Black, NH	205.73	266.96	3.44	2.79
	Hispanic	237.22	250.30	1.06	1.42
	White, NH	198.56	234.77	3.53	3.20

Note: Cases and death rates per 100,000. Mississippi data only source was from the MSDH; 59.81/100,000 cases and 0.53/100,000 deaths regard unknown racial or ethnic identity. AI: American Indian, AN: Alaska Native, NH: Non-Hispanic, Pl: Pacific Islander.

**Table 4:** Rates of socioeconomic status variables and the percent of racial populations.

State	Poverty Rate	Uninsured Rate	Medicaid Insurance	Unemployment	High School Education	College Education	White	Black	Hispanic	Asian	American Indian/ Alaska Native
AL	15.03	9.7	19.5	2.60	35.9	16.1	60.10	12.20	18.50	5.60	0.70
FL	12.56	13.1	17.4	2.70	39.2	15.1	65.40	26.50	4.40%	1.40	0.40
GA	13.39	13.4	17.3	2.80	36.7	17.3	53.00	15.00	26.60	2.70	0.20
LA	19.05	8.9	29.3	3.50	40.8	15.4	52.00	31.30	9.80%	4.10	0.20
MS	19.07	12.9	24.2	3.60	32.5	18.9	58.80	31.90	5.20%	1.70	0.50
TN	13.74	10.2	19.5	3.40	41.7	16.3	56.60	37.60	3.00	1.00	0.40
USA	12.3	9.2	19.8	3.5	35.4	20.7	73.60	16.40	5.70	1.80	0.30

## Data Availability

Not applicable.
